# A Bill to Provide for Registration in New South Wales

**Published:** 1899-06

**Authors:** 


### SELECTIONS.

### A Bill to Provide for Registration in New South Wales.

Another proposed measure, described as a "A Bill to Provide for the Registration of Dentists Qualified to Practice in New South Wales," is very much on all fours with the Victorian Act. The sections providing for the qualification necessary for registration are as follows:

11. Any person who—(*a*) is registered in the United Kingdom in accordance with the laws for the time being in force therein as a dentist or medical practitioner; or (*b*) is entitled as hereinafter mentioned to be registered under this Act as a dentist; or (*c*) has for a period of six months before the commencement of this Act been *bona fide* engaged in New South Wales in the practice of dentistry, either separately or in conjunction with the practice of medicine, surgery, or pharmacy, and who has made application for registration to the Board within one year from the commencement of this Act; or (*d*) has attained the age of twenty-one years and has been engaged during a period of not less than four years in the acquirement of professional knowledge in dentistry, and has passed an examination before the Board according to the prescribed regulations; or (*e*) has attained the age of twenty-one years, and shall have been a pupil or apprentice of a registered dentist for a period of two years, and shall have been such pupil or apprentice for a period of six months before the commencement of this Act; or (*f*) has obtained a diploma or degree in dentistry from a University in Australia shall be entitled to be registered as a dentist under this Act.

12. Any person who has practised dentistry for not less than twelve months elsewhere than in New South Wales, and who holds some recognized certificate as hereinafter defined, and who proves to the satisfaction of the Board that he is of good character, shall be entitled, upon the payment of the prescribed registration fees, and without examination, to be registered as a dentist under this Act. The term "recognized certificate" means a certificate, diploma, membership, degree, license, letters, testimonial, or other title, status, or document granted by some university, college, or other public institution in a British possession or foreign country, and which is recognized by the Board as entitling the holder thereof to practice dentistry in such possession or country, and as furnishing sufficient guarantee of the possession of the requisite knowledge and skill for the efficient practice of dentistry.

The Board for the first three years is to be appointed by the Government; after this to be elected by the dentists.

